# Development of the Comprehensive General Parenting Questionnaire for caregivers of 5-13 year olds

**DOI:** 10.1186/1479-5868-11-15

**Published:** 2014-02-10

**Authors:** Ester FC Sleddens, Teresia M O’Connor, Kathleen B Watson, Sheryl O Hughes, Thomas G Power, Carel Thijs, Nanne K De Vries, Stef PJ Kremers

**Affiliations:** 1 Maastricht University, Department of Health Promotion, NUTRIM School for Nutrition, Toxicology and Metabolism, Maastricht University Medical Center+, P.O. Box 616, 6200, MD Maastricht, The Netherlands; 2 Department of Pediatrics, Baylor College of Medicine/USDA Children’s Nutrition Research Center, 1100 Bates Street, Houston, TX 77030, USA; 3 Department of Human Development, Washington State University, P.O. Box 644852, Pullman, WA 99164-4852, USA; 4 Maastricht University, Department of Epidemiology, CAPHRI School of Public Health and Primary Care, P.O. Box 616, 6200, MD Maastricht, The Netherlands; 5 Maastricht University, Department of Health Promotion, CAPHRI School of Public Health and Primary Care, NUTRIM School for Nutrition, Toxicology and Metabolism, Maastricht University Medical Center+, P.O. Box 616, 6200, MD Maastricht, The Netherlands

**Keywords:** Body mass index, Children, General parenting, Questionnaire development, Parent personality, Validation

## Abstract

**Background:**

Despite the large number of parenting questionnaires, considerable disagreement exists about how to best assess parenting. Most of the instruments only assess limited aspects of parenting. To overcome this shortcoming, the “Comprehensive General Parenting Questionnaire” (CGPQ) was systematically developed. Such a measure is frequently requested in the area of childhood overweight.

**Methods:**

First, an item bank of existing parenting measures was created assessing five key parenting constructs that have been identified across multiple theoretical approaches to parenting (Nurturance, Overprotection, Coercive control, Behavioral control, and Structure). Caregivers of 5- to 13-year-olds were asked to complete the online survey in the Netherlands (N = 821), Belgium (N = 435) and the United States (N = 241). In addition, a questionnaire regarding personality characteristics (“Big Five”) of the caregiver was administered and parents were asked to report about their child’s height and weight. Factor analyses and Item-Response Modeling (IRM) techniques were used to assess the underlying parenting constructs and for item reduction. Correlation analyses were performed to assess the relations between general parenting and personality of the caregivers, adjusting for socio-economic status (SES) indicators, to establish criterion validity. Multivariate linear regressions were performed to examine the associations of SES indicators and parenting with child BMI z-scores. Additionally, we assessed whether scores on the parenting constructs and child BMI z-scores differed depending on SES indicators.

**Results:**

The reduced questionnaire (62 items) revealed acceptable fit of our parenting model and acceptable IRM item fit statistics. Caregiver personality was related as hypothesized with the GCPQ parenting constructs. While correcting for SES, overprotection was positively related to child BMI. The negative relationship between structure and BMI was borderline significant. Parents with a high level of education were less likely to use overly forms of controlling parenting (i.e., coercive control and overprotection) and more likely to have children with lower BMI. Based on several author review meetings and cognitive interviews the questionnaire was further modified to an 85-item questionnaire.

**Conclusions:**

The GCPQ may facilitate research exploring how parenting influences children’s weight-related behaviors. The contextual influence of general parenting is likely to be more profound than its direct relationship with weight status.

## Background

Parents are critical in influencing children’s health behaviors and subsequently their weight status. For instance, they are the gatekeepers of the home food supply and responsible for providing access to regular physical activity. Through the use of parenting practices, defined as context-specific acts of parenting [1] to socialize children toward certain behavior, such as completing homework or chores, parents can have an influence on a wide range of health behaviors. Specific parenting practices related to food (e.g., using food as a reward, educating their child about importance of healthy eating) and physical activity (e.g., parental support to be physically active or parents being physical active in front of the child) have been demonstrated to be associated with weight-related behavior in children, although study methodology and findings varied considerably (e.g., [2-4]). Parents also influence their child’s behavior through the use of general parenting styles, the larger context in which these parenting practices are expressed creating the emotional climate within which practices can be accepted or rejected by the child. In a review by Sleddens, Gerards, Thijs, De Vries, and Kremers [5], authoritative forms of general parenting were found to be positively related with healthy eating, physical activity, and lower Body Mass Index (BMI) levels among children; although these relationships were generally weak and some conflicting findings were reported. Accumulating evidence shows that the moderating role of general parenting on the relationship between more specific parenting practices and weight status or related behavior is likely to be more profound than its direct relationship with weight-related outcomes [5].

Current interventions to prevent childhood overweight and obesity have been largely ineffective due to a lack of understanding of how parents influence child behaviors [6,7]. Childhood interventions with the best outcomes (i.e., increased healthy eating and physical activity) have engaged parents [8] and interventions targeting specific parenting practices have been shown to benefit from being tailored to the home’s emotional general parenting climate [9]. In addition, in order to develop effective interventions, the exact mechanisms of parenting on children’s weight-related outcomes need to be unraveled.

Inconsistent findings about the role of parents on children’s weight-related outcomes are likely due to the large diversity of instruments to assess parenting in this respect [5]. We found that more than 20 different instruments were used in studies relating general parenting to children’s weight-related outcomes. Therefore, it is difficult to compare findings between different studies. Furthermore, differences in conceptualization of parenting constructs may also explain inconsistent findings. Despite the large number of general parenting instruments available [10,11], considerable disagreement exists about how to best assess parenting. Most of the instruments only assessed limited aspects of parenting, and consensually identified questionnaires of high quality measures are lacking [5]. Hence, it is necessary to identify the core constructs of parenting and to elaborate and clarify their defining features. We decided to develop a comprehensive general parenting questionnaire for widespread use.

Developing a single parenting questionnaire to assess the major parenting constructs (versus piecing together a large number of individual questionnaires) greatly reduces participant response burden. Moreover, by measuring the major parenting dimensions simultaneously, it will be possible in future studies to examine individual differences in parenting styles that involve simultaneously assessing individuals across multiple parenting dimensions. The ultimate goal is to promote comparison across studies and facilitate research exploring how parenting influences children’s weight-related behaviors.

### Theoretical approaches to general parenting

General parenting has commonly been defined as the approach parents use to raise their child, and are a function of the parent’s attitudes, beliefs and behaviors, creating a family emotional climate [1,12,13]. Parenting is a complex interplay of specific behaviors intended to influence child outcomes, and displayed across many different situations [1]. Parenting has been examined from a variety of theoretical perspectives including psychoanalytic [14], operant learning [15,16], social learning [17,18], acceptance-rejection [19], attachment [20,21], self-determination [22], and Vygotskian [23] theories. In contrast to early investigations that examined the child development consequences of specific parenting practices (e.g., the nature and timing of weaning or toilet training) [14], most theoretical approaches (operant and Vygotskian approaches being notable exceptions) have led to studies examining the child development correlates of cross-situational variations in general parenting approach—often referred to as parenting *styles* or *dimensions*. These studies focused less on *what* parents do and more on *how* they do it. Skinner, Johnson, and Snyder [24], in a review of this literature, showed that independent of theoretical perspective, most researchers have focused on three core dimensions of parenting practices (warmth versus rejection, structure versus chaos, and autonomy support versus coercion). These are the three dimensions we focused on in the development of our instrument, referred to below as parental nurturance, structure, and control.

### Toward a comprehensive assessment of general parenting

Although there is considerable convergence across studies on the child development correlates of parental nurturance and structure [24], the literature on parental control is much less consistent [25,26]. While nurturance and structure are well defined in the parenting literature, multiple forms of control have been identified by several scientists, some inhibiting and others supporting a child’s emotional development. Some controlling practices are thought to support children’s development, such as developmentally appropriate forms of guidance and direction (also called *behavioral control*). Other forms of control appear to inhibit children’s development include parental strictness, excessive parental involvement or worry (*overprotection*), and parental dominance or intrusiveness (*coercive control*).

**Figure 1:**
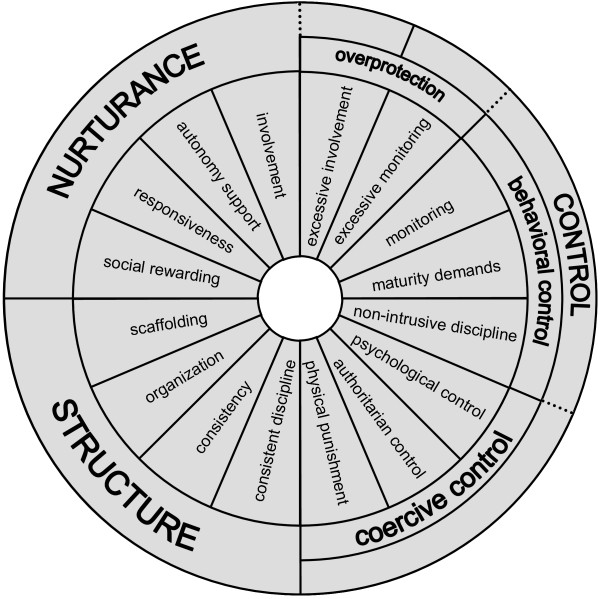
**Comprehensive general parenting model.***Note*: Five-factor parenting model for the development of the Comprehensive General Parenting Questionnaire.

Thus, we identified five parenting constructs (i.e., *nurturance, structure, behavioral control, overprotection, and coercive control*) that describe the major individual differences in general parenting behavior. Each of these constructs will be clarified in the following sections. Figure 1 displays our comprehensive general parenting model.

#### Nurturance

This is one of the parenting constructs most frequently assessed. Nurturance represents the degree to which parents foster and recognize individuality and self-assertion by being supportive and responsive to their child’s needs, showing interest in child activities, spending time with their child, praising their child for good behavior, and expressing affection and care (warmth; [19]) toward their child. The literature supports four sub-constructs that encompass nurturance. These include “responsiveness” (the extent to which parents are aware of their child’s feelings, problems, and difficulties, and the way they respond in a supportive and attuned manner), “autonomy support” (parenting behaviors in which children are promoted to express their feelings and opinions; e.g., [22,27]), “social rewarding” (verbally praising their child as a reward for good behavior), and “involvement” (parents being involved with their child by attending the child’s events and activities, and spending time with their child).

#### Structure

It is the degree to which parents organize their child’s environment, by helping their child when necessary to gradually achieve a certain goal, and consistently enforcing rules and boundaries. Sub-constructs include “inconsistent discipline” (reverse coded; parents scoring high on concepts such as non-contingency and inconsistency, acting erratic, unpredictable and undependable, and not following through when disciplining their child), “consistency” (acting in a predictable manner by providing and explaining clear and consistent guidelines, enforcing those rules, and keeping promises to their child; [28]), “organization” (helping their child to organize regular activities; e.g., [28,29]), and “scaffolding” (exposing children to activities that foster the development of new skills and providing just enough structure and assistance to help them solve problems and learn with the ultimate goal of enabling children to perform the task independently; [23,30]).

#### Behavioral control

This construct could be regarded as parents supervising and managing their child’s activities, providing clear expectations for behavior (in this paper referred to as maturity demands), and using disciplinary approaches in a non-intrusive manner. Parents scoring high on behavioral control provide adequate levels of control, are not too strict or over-controlling, but rather allow their child to have enough space to develop independence and autonomy. As Darling and Steinberg [1] formulated in 1993, it refers to the parent’s “willingness to act as a socializing agent”. The identified sub-constructs are as follows: “monitoring” (supervising their child’s activities) and “maturity demands” (expectations for behavior) [31], and “non-intrusive discipline” (the use of disciplinary approaches when children misbehave that are mainly based on explaining a child’s misbehaviors, taking away privileges and correcting the child in a non-intrusive manner).

#### Overprotection

One of the most understudied aspects of parental control is parental overprotection [32]. With a few notable exceptions (e.g., [33,34]), most of what has been written about the negative effects of parental overprotection come from clinical case studies (e.g., [35,36]) or from media reports of “helicopter parents”. Parents who are overprotective score high on “excessive involvement” (excessive nurturing) and “excessive monitoring” (strict control). They are believed to negatively impact child development through interfering with the development of children’s autonomy. Although it is difficult to describe for a given child what constitutes “excessive” involvement or monitoring, it is defined here as involvement or monitoring that is excessive given the child’s developmental level. Therefore, if a parent shows a level of involvement or monitoring that is more appropriate for a much younger child, it is viewed as excessive. Because this newer construct was not specifically addressed in the Skinner et al. [24] model, we are including it under the control construct. The sub-construct of “excessive involvement” is defined as being too involved with their child (parents being overprotective by not letting their child get involved in activities if there is a slight chance to fail, and spending every free minute they have with their children). “Excessive monitoring” is defined as parents who excessively monitor their child’s behavior (characterized by overprotective parental behaviors such as frequently checking where the child is and what the child is doing, more than is considered appropriate for the child’s age).

#### Coercive control

We refer to coercive control as parents characterized by pressure, intrusion, domination, and discouragement of child independence and individuality. The sub-constructs of this parenting construct are “authoritarian control” (parents who tend to enforce rules harshly, expect their child to accept their judgments, values, and goals without questioning, and attempt to control their child’s emotions at all times; Baumrind [12,13]), “physical punishment” (using corporal punishment as a way of disciplining the child), and “psychological control” (parental behaviors that are intrusive and manipulative of children’s thoughts, feelings, attachments to parents ([37], p. 15). Psychological control intrudes into the psychological and emotional development of the child through use of parenting practices such as guilt and anxiety induction, love withdrawal, constraining verbal expressions, and personal attacks on a child [25]. It was first defined by Becker [38] in 1964 as negative, love-oriented discipline such as child isolation from the parent and love withdrawal. Schaefer’s work [39,40] included psychological control as the presence of parental dominance, intrusiveness, and coercive, autocratic discipline. From the 70s to the 90s, the construct of psychological control was largely neglected in empirical research on the socialization process, because in these decades the typological approaches to parenting focusing on the “responsiveness” and “demandingness” dimensions dominated the socialization literature [41,42]. After this period, Steinberg [43] and Barber [25,44] re-focused on the construct of psychological control.

### Establishing criterion validity

This study aimed to develop and validate a new “Comprehensive General Parenting Questionnaire” (CGPQ) to assess the five key constructs of parenting reviewed above.

#### Relationship with adult personality

Parenting is influenced by numerous facets of the caregiver; one of the main determinants is parent personality [45]. Assessment of personality is commonly based on five-factor taxonomy of traits, the so-called “Big Five”, which has proven very useful for conceptualizing and measuring individual differences in personality (e.g., [46-48]). Consensus has been achieved concerning the five-factor personality structure as it has been proven to replicate in diverse samples, across languages and cultures, and across several assessment methods and factor analytic procedures [49]. The “Big-Five” factors have been labeled as follows: (1) extraversion, (2) agreeableness, (3) conscientiousness, (4) openness to experience (or intellect, culture), and (5) neuroticism (vs. emotional stability) [50]. Within the parenting literature, a meta-analytic review was previously conducted examining links between the “Big Five” personality factors and parenting [51]. Findings showed that higher levels of extraversion, agreeableness, conscientiousness, and openness to experience and lower levels of neuroticism were related to more parental warmth and behavioral control, whereas only higher levels of agreeableness and lower levels of neuroticism were related to more autonomy support. Neuroticism has been repeatedly found to be associated with less adaptive parenting behaviors. In the current study, relations between general parenting and personality characteristics of the caregivers were assessed as a measure of construct validity as child-rearing varies depending on parent personality. Based on previous findings [51] we expected that caregivers who score high on “positive” parenting (i.e., nurturance, structure, and behavioral control) would also score high on the more positive related personality traits including agreeableness, extraversion, conscientiousness, openness to experience, and score low on neuroticism.

#### Relationship with child body mass index

In the current study, we were interested in examining whether the results of previous studies assessing the parenting style and child overweight relationship [5] would be replicated, using the standardized child BMI scores (BMI z-scores) as an outcome. We hypothesized the more positive forms of parenting to be related to lower BMI z-scores in children, whereas the other forms of parenting (i.e., coercive control and overprotection) would be related to higher BMI z-scores in children. However, these relations are likely relatively weak as general parenting operates as a more distal predictor of childhood weight-related outcomes than more proximal behavior-specific parenting practices [5,52]. Previous studies also suggest that general parenting acts as a moderator on the influence of these specific parenting practices [5].

## Methods

A mixed methods approach was used to develop the CGPQ comprising of the following four steps: (1) Items were identified from existing parenting questionnaires based on our framework including the five constructs of parenting. (2) Cognitive interviews and author review informed the modification, deletion and/or replacement of items. (3) Advanced statistical analyses including Classical Test Theory, Confirmatory Factor Analyses (CFA) and Item-Response Modeling (IRM) were conducted to test our theoretical five-factor parenting model and to develop fit items using an online survey containing the parenting item bank. (4) Finally, additional author reviews and cognitive interviews were done to review the fit items, determine if any construct was missing or inadequately assessed, assess content validity, and verify wording of the modified items.

### Scale development

We searched for validated instruments measuring our defined parenting constructs (see Figure 1), and selected some of the most commonly used instrument in research. An item bank was created by pulling and adapting items from the following existing questionnaires: the “Parents as Social Context Questionnaire” [24]; the “Ghent Parental Behavior Scale” [53]; the “Child Rearing Practices Report” [54,55]; the “Parenting Dimensions Inventory” [28,56]; the “Parental Regulation Scale – youth self-report: parental expectations for behavior scale and parental monitoring of behavior scale” [57,58]; the “Psychological Control Scale – youth self-report” [25,58] and its adaptations to parent self-reported parenting [59,60]; the “Parental Authority Questionnaire” [61]; the Dutch Parenting Questionnaire (‘Nijmeegse Opvoedingsvragenlijst’; [62]), and the “Perceptions of Parents Scales” [63-65]. Team meetings were held to ensure face validity of the items, and modifications were made to improve ambiguous items. In case of unavailability of the measures in both Dutch and English, the items of concern were translated by the first author, a Dutch native speaker and fluent speaker of the English language, and cross-checked by the co-authors. All authors approved the final English translations. Cognitive interviewing was conducted on several of these questionnaires [24,55,60] with 10 to 20 Dutch parents to ensure that they understood the items and response scales. This pre-test consisted of parents completing the questionnaire, followed by discussion of particular words/phrases to see whether parents understood the items as intended, and discussion of items parents identified as complex. For the interview a pre-defined interview script was used. Minor changes were made in wording. Moreover, based on an in-depth review of existing parenting literature and validated measures, we wrote additional items to provide adequate number of items to cover all sub-constructs of the five different parenting constructs. The resulting questionnaire included 145 items that measured nurturance, structure, behavioral control, overprotection, and coercive control. For all items the same 5-point Likert scale was used, ranging from 1 (*strongly disagree*) to 5 (*strongly agree*).

### Additional measures

In addition to the parenting questions, we collected parent-reported demographic information (e.g., child gender, age, and height and weight, living situation (coded as 1 = living together with child and spouse; 2 = living together with child no spouse; 3 = other), parental education level (1 = low; 2 = medium; 3 = high) and employment status (1 = unemployed, not having paid job; 2 = employed, having paid job), see Table 1). Caregiver’s personality was measured using a 30-item scale for the “Big Five” (six for each of the traits) [66]. The criterion validity, test–retest reliability and internal consistency of this 30-item scale have been well established in previous studies [67]. Caregivers were asked to score on a 7-point Likert scale the degree to which the personality characteristics were descriptive of themselves. Cronbach’s alphas were as follows: extraversion 0.88, agreeableness 0.85, conscientiousness 0.88, openness to experience 0.80, neuroticism 0.81. Using children’s height and weight data, BMI of the children was calculated and converted to a standardized z-score, adjusting for age and gender, based on reference data of the World Health Organization (WHO) growth reference [68]. BMI z-scores < -5.0 or > 5.0 were considered unrealistic and removed as advised by the WHO [68].

**Table 1:** Sample characteristics

		**Netherlands (N = 821)**		**Belgium (N = 435)**		**U.S. (N = 241)**	
**Description**		***n***	**%**	***n***	**%**	***n***	**%**
*Child gender*	Male	408	49.7	213	49.0	128	53.1
	Female	413	50.3	222	51.0	113	46.9
*Relationship to child*	Female caregiver	519	63.2	336	77.2	203	84.2
	Male caregiver	302	36.8	99	22.8	38	15.8
*Race (US only)*	Black or African-American					46	19.1
	White or Euro-American					112	46.5
	Hispanic or Latino					59	24.4
	Other					24	10.0
*Living situation*	Together with child and spouse	727	88.6	377	86.7	186	77.2
	Together with child and no spouse	84	10.2	56	12.9	43	17.8
	Other	10	1.2	2	0.5	12	5.0
*Education*^*a*^	Low	127	15.5	21	4.8	13	5.4
	Medium	305	37.1	61	14.0	88	36.5
	High	389	47.4	353	81.1	140	58.1
*Employed: paid job*	Yes	718	87.5	393	90.3	200	83.0
	36 hours or more per week	290	40.4	210	53.4	167	83.5
	20 to 35 hours per week	275	38.3	144	36.6	18	9.0
	12 to 19 hours per week	105	14.6	35	8.9	10	5.0
	Less than 12 hours per week	48	6.7	4	1.0	5	2.5
	No	103	12.5	42	9.7	41	17.0

*Note:*^a^Highest education attained, categorized into low level (8^th^ grade or less, attended some high school, technical school graduate), medium level (high school graduate or general educational development, some college), and high level (college graduate, post graduate study).

### Data collection and participants

The survey was administered as a web-based survey which has more advantages than disadvantages compared with traditional modes of data collection. Advantages include lower proneness to social desirability bias, no missing data when using forced-choice formats, and more rapid return than postal questionnaires [69]. Disadvantages include selection bias for those that have access to a computer, and higher non-response rates, although subjects responding to an online survey are comparable to those responding to traditional modes of data collection in terms of demographics [69].

#### The Netherlands

Data were collected using a random sample of eligible parents (i.e., caregivers of 5- to 13-year-olds) from two Dutch Internet survey panels (Flycatcher Internet Research BV and Thesistools). The companies performed the random selections, ensuring the sample remained representative of the countries. Participants who take part in the Flycatcher panel are financially rewarded for their contribution, e.g. by collecting points for every completed questionnaire in order to be able to receive a gift coupon after a number of questionnaires. Only participants who had completed all parenting items were used for the current study. In total, 517 questionnaires were completed via Flycatcher and 304 via Thesistools. Child mean (*SD*) age was 8.64 (2.00) years.

#### Belgium

Similar procedures were used to generate data from Belgian parents. A Dutch Internet panel, Thesistools, was used for distribution of our online survey to eligible Dutch speaking parents in Belgium. In total, 435 questionnaires were used for analysis. Child mean (*SD*) age was 9.43 (1.88) years.

#### United States

In the United States, English-speaking parents were informed about the online survey by (a) posting and handing out flyers in the vicinity of the Texas Medical Center, community centers, public libraries, universities, sports centers, and museums throughout Houston, Texas; (b) posting the study on the website of Baylor College of Medicine and the Children’s Nutrition Research Center (CNRC); and (c) listing the study in the CNRC’s nationally distributed newsletter and recruiting from the CNRC participant database. From all completed entries (N = 241), three names from the U.S. sample were randomly selected to receive a $50 gift card. Only participants who agreed to take part in the raffles had a chance to win one of the gift cards. Child mean (*SD*) age was 9.18 (2.26) years.

### Data analysis

Based on several author review meetings with some of the leading researchers from the parenting field having extensive experience in questionnaire item development (based on qualitative and advanced statistical methods), 30 items were dropped prior to data analysis from the list of 145 parenting items. These items were dropped because of redundancy of item content or ambiguity. Data reduction procedures (i.e., CFA and IRM) were used to further reduce the list of 115 items on the total sample of parents (N = 1497). The use of the total sample provided adequate power to perform the data reduction procedures on the list of 115 items. Table 2 gives an indication of the number of items within each of the five parenting constructs and the corresponding sub-constructs.

**Table 2:** Comprehensive General Parenting Questionnaire average scores and item separation reliability

**Parenting constructs**	**Mean ( *SD *)**	**EAP/PV reliability**
*Nurturance (19 items)*	*4.46 (0.40)*	*0.86*
Responsiveness (6 items)	4.48 (0.47)	0.79
Autonomy support (5 items)	4.51 (0.47)	0.73
Involvement (4 items)	4.22 (0.64)	0.79
Social rewarding (4 items)	4.63 (0.46)	0.75
*Structure (15 items)*	*3.84 (0.45)*	*0.75*
Inconsistent discipline (3 items)	2.90 (0.86)	0.73
Consistency (5 items)	4.47 (0.52)	0.69
Organization (3 items)	3.73 (0.89)	0.74
Scaffolding (4 items)	4.64 (0.41)	0.67
*Behavioral control (10 items)*	*4.00 (0.49)*	*0.69*
Monitoring (3 items)	4.02 (0.73)	0.68
Maturity demands (5 items)	4.31 (0.52)	0.75
Non-intrusive discipline (2 items)	3.19 (1.00)	0.33
*Overprotection (6 items)*	*2.55 (0.55)*	*0.53*
Excessive monitoring (2 items)	3.31 (0.73)	0.49
Excessive involvement (4 items)	2.17 (0.63)	0.52
*Coercive control (12 items)*	*2.06 (0.50)*	*0.75*
Psychological control (5 items)	1.84 (0.66)	0.71
Physical punishment (3 items)	1.34 (0.58)	0.62
Authoritarian control (4 items)	2.87 (0.69)	0.66

*Note:* Number of questionnaire items = 62 (following CFA and IRM analyses).

A second-order CFA was used to validate the hypothesized five-factor structure (nurturance, structure, behavioral control, overprotection, and coercive control). The second-order model allowed for sub-constructs loading onto the higher order constructs. In the first model we constrained the parenting factors so they did not correlate, whereas in the second model they were allowed to correlate. Given that the data were not severely skewed or kurtosed, parameter estimates were obtained using the maximum likelihood estimation procedure. Items were dropped that did not fit the model (i.e., with factor loadings equal or less than 0.40). The chi-square goodness-of-fit test and three fit indices were used to assess model fit, including the Root Mean Square Error of Approximation (RMSEA), the Comparative Fit Index (CFI), and the Non-Normed Fit Index (NNFI). Criteria of Hu and Bentler [70] were used to evaluate model fit: RMSEA with a value of ≤ 0.05 indicating a good fit and an upper value of 0.08 representing a reasonable fit; CFI and NNFI with a value > 0.95 indicating a good fit.

Rasch Modeling (Multidimensional Partial Credit Model) was employed to further assess the psychometric properties of the parenting questionnaire and to reduce items, using the ConQuest software [71]. These analyses were performed on the five parenting constructs separately, allowing us to incorporate the multidimensionality of sub-constructs within parenting constructs. The IRM analyses yield item infit statistics, item parameter difficulty estimates, Wright maps, and reliability indices. Item fit was determined by computing the weighted mean square fit statistics for each item, which indicate whether residuals varied as much as expected given the observed distribution. Items with a weighted infit statistic between 0.75 and 1.33 and/or items with a corresponding weighted *t*-statistic between -2.00 and 2.00 were indicative of a good fit [72]. Examination of item fit was the first step in removing items using IRM.

The next step was to identify items with overlapping levels of item average difficulty via the Wright map. In the context of general parenting, item difficulty refers to the level of agreement in performing the parenting practices. Item difficulty is the item’s location on the underlying parenting construct, a “higher” location indicating an increment in level of difficulty for the respondent to answer more agreeably to an item. Among items with overlapping levels of difficulty, item removal decisions were based on several meetings with the research group ensuring content validity was not threatened. Item separation reliability (EAP/PV) was calculated for the parenting scales’ underlying the parenting constructs. It indicated “how well the sample of subjects had spread the items along the measure of the test” ([73], p. 238). The EAP/PV reliability is analogous to Cronbach’s alpha and can be interpreted similarly where the minimum acceptable cut-off level for Cronbach’s alpha is 0.50 [74].

Mean factor scores were computed for the five constructs of the CGPQ (see Table 2) and the “Big Five” personality questionnaire (see Table 3). Correlation coefficients were used to assess associations between the scores for the five parenting constructs and to assess the associations between the scores for the parenting constructs and the “Big Five” personality constructs, partialling out the effects from child gender and age, parental education level (ranging from 1: lowest level of education, to 3: highest level of education), and parental employment status (dichotomized as 1: unemployed or 2: employed). The strength of the relationship between the variables studied was assessed using correlation effect sizes as suggested by Cohen [75] with respect to partial correlations: small (0.02 - 0.15), medium (0.15 - 0.35), and large (0.35 - 1.0).

**Table 3:** Correlations between the five general parenting constructs and parent personality and child BMI z-scores

**Measure**		**Mean ( *SD *)**	**Nurturance**	**Structure**	**Behavioral control**	**Overprotection**	**Coercive control**
*Big Five*	Extraversion	5.19 (1.26)	0.27**	0.24**	0.08**	−0.09**	−0.17**
	Agreeableness	5.86 (0.73)	0.42*	0.31**	0.14**	0.02	−0.21**
	Conscientiousness	5.06 (1.19)	0.14**	0.35**	0.18**	0.13**	0.02
	Openness to experience	4.78 (1.11)	0.29**	0.21**	0.06*	−0.06*	−0.18**
	Neuroticism	3.25 (1.16)	−0.20**	-0.30**	0.05*	0.18**	0.32**
*Parenting*	Nurturance	4.46 (0.40)	-	-	-	-	-
	Structure	3.84 (0.45)	0.49**	-	-	-	-
	Behavioral control	4.00 (0.49)	0.33**	0.18**	-	-	-
	Overprotection	2.55 (0.55)	0.01	−0.06*	0.22**	-	-
	Coercive control	2.06 (0.50)	−0.37**	−0.32**	0.27**	0.37**	-

*Note*: *n* = 1482; missing parent personality *n* = 4; missing child age *n* = 11; 62-item parenting questionnaire; *p ≤ 0.05 (two-sided), **p ≤ 0.01 (two-sided).

Multivariate linear regression analyses were performed to assess the contribution of socio-economic status (SES) indicators (i.e., parental educational level and employment status) and the five parenting constructs on child BMI z-scores. All predictor variables were entered simultaneously into the equation. Additionally, we excluded underweight children with a BMI z-score below -1.0 to prevent distortion of the results (cf. [76]). Furthermore, we assessed whether scores on the general parenting constructs and child BMI z-scores differed depending on SES indicators (i.e., parental educational level and employment status).

## Results

### Sample characteristics

Characteristics of the study samples are depicted in Table 1. Most often, female caregivers completed the online survey. Child gender was nearly equally divided across the three samples. Most caregivers indicated they lived with the child and spouse (percentages ranging from 77.2% in the U.S. to 88.6% in the Netherlands). The U.S. study sample was ethnically diverse. The majority was White (46.5%), but Hispanics (24.4%) and African-Americans (19.1%) were also represented. A minority of the US participants were combined into “other”, consisting of American Indians, Native Hawaiians, Pacific Islanders, and Asians (10.0%). A large percentage of participants from the Netherlands, Belgium and the U.S. reported higher levels of education (47.4%, 81.1%, and 58.1%, respectively, indicated having a college degree or higher) and were employed (87.5%, 90.3%, and 83.0%, respectively). Our study populations were roughly representative samples of the Dutch, Belgian and U.S. population. Compared to the general U.S. population, whites were underrepresented in the current study (46.5%), but our U.S. sample had a demographic distribution (i.e., ethnically diverse sample) similar to the Houston population. Participants with higher levels of education were slightly overrepresented in the current samples, but employment rates were largely similar to the general populations. Valid parental reported weight and height of their children was available for 1015 respondents. The mean BMI z-score was -0.13 (*SD* = 1.40) for the total sample. In total, 260 children were underweight (BMI z-score < -1.0).

### Confirmatory factor analysis

CFA revealed a relatively adequate fit of our hypothesized general parenting model (X^2^ = 26606.39, df = 6418, p < 0.001; RMSEA = 0.06, CFI = 0.91, NNFI = 0.91) when the parenting constructs were not allowed to correlate. The fit slightly improved after allowing the parenting constructs to correlate (i.e., X^2^ = 25434.68, df = 6414, p < 0.001; RMSEA = 0.06, CFI = 0.92, NNFI = 0.92). Subsequently, 33 items were removed based on the following criteria: magnitude of loadings (e.g., <0.40), contribution to construct coverage, and theoretical considerations. The reduced 82-item model had a slightly better fit compared to the 115-item model (parenting constructs not allowed to correlate: X^2^ = 14013.87, df = 3217, p < 0.001; RMSEA = 0.05, CFI = 0.93, NNFI = 0.92; parenting constructs allowed to correlate: X^2^ = 12864.61, df = 3213, p < 0.001; RMSEA = 0.05, CFI = 0.93, NNFI = 0.93).

### Item-response modeling

IRM analyses on each of the five parenting constructs using multidimensional models indicated that all 82-items had acceptable values for both the weighted mean square statistic and *t* statistic. To further reduce the number of items in the questionnaire, the Wright maps were visually inspected to assess overlapping item coverage across the latent parenting factors. Subsequently, 20 items were removed, until the total number of items per parenting sub-construct was around five based on the following criteria: items with overlapping levels of difficulty, contribution to construct coverage, and theoretical considerations. Thereafter, IRM was repeated on the reduced set of items (62 items in total) for each of the five parenting constructs. All items had acceptable values for both the weighted mean square statistic and *t* statistic (range of infit statistics, *t* statistic between brackets: nurturance 0.85 (-2.0) – 1.26 (4.9), structure 0.86 (-4.4) – 1.17 (5.0), behavioral control 0.88 (-2.8) – 1.17 (3.7), overprotection 0.98 (-0.5) – 1.05 (0.9), and coercive control 0.91 (-1.6) – 1.21 (3.3)). Item difficulty estimates (*SE*) ranged from -0.84 (0.04) to 0.64 (0.05) for nurturance, from -0.56 (0.02) to 0.67 (0.02) for structure, from -0.58 (0.03) to 0.77 (0.03) for behavioral control, from -1.24 (0.02) to 1.24 (0.02) for overprotection, and from -0.96 (0.02) to 0.77 (0.04) for coercive control. Based on the Wright map, the items of the parenting constructs of nurturance, structure, and behavioral control covered a restricted portion of participants (only those scoring low on this factor) in that the upper end of the continuum remained uncovered by items with higher levels of difficulty. The reverse was seen for the other two parenting constructs of coercive control and overprotection. EAP/PV reliability estimates slightly dropped for the several parenting constructs as expected, most likely due to item removal (ranged between 0.52 and 0.86). We refer to Table 2 for an overview of the number of items per parenting sub-construct and the reliability estimates.

### Associations between parenting and caregiver personality

Associations between the parenting constructs on the reduced 62-item questionnaire were as follows (see Table 3): nurturance, structure and behavioral control were positively intercorrelated as well as the constructs of overprotection and coercive control, with small to medium effect sizes. Additionally, both nurturance and structure were positively related with behavioral control and negatively related with coercive control. The negative relationship with overprotection was only significant for structure, not for nurturance. Behavioral control on the other hand was positively related with overprotection and coercive control (small effect sizes).

Associations between the five parenting constructs on the reduced 62-item questionnaire and “Big Five” personality characteristics of the caregivers are also reported in Table 3. Positive correlations (small to medium effect sizes) were found for the association between the four features of the “Big Five” (i.e., extraversion, agreeableness, conscientiousness, and openness to experience) and the three positive parenting constructs (i.e., nurturance, structure, and behavioral control). These personality characteristics tended to be negatively correlated with coercive control and overprotection. However, conscientiousness was positively associated with overprotection and not associated with coercive control, and agreeableness was not associated with overprotection. For the personality characteristic of neuroticism, negative correlations with nurturance and structure were found, whereas positive correlations were found with behavioral control, coercive control and overprotection (small to medium effect sizes).

### The contribution of parenting dimensions and SES indicators to child BMI

Multivariate linear regression analyses were performed to assess the contribution of both SES indicators (i.e., parental educational level and employment status) and the five parenting constructs on child BMI z-scores. The multivariate model in Table 4 showed that after correction for SES indicators, overprotection was significantly and positively associated with child BMI z-score for the total sample of children with valid BMI data (*ß* = 0.083, p < 0.05) and for the total sample excluding the underweight children (*ß* = 0.091, p < 0.05). Structure was only marginally related to child BMI z-score for the total sample of children with valid BMI data (*ß* = -0.072, p = 0.051). Of the two SES indicators, only parental education level was associated with child BMI z-score. This association was significant and negative for the two samples (*ß* = -0.096, p < 0.01 for the total sample, *ß* = -0.106, p < 0.01 for the sample excluding underweight children).

**Table 4:** Association of SES indicators and general parenting with child BMI z-scores

		**Regression coefficient ( *ß *)**	
		**Child BMI z-score (*n*** **= 1015)**	**Child BMI z-score (*n*** **= 755)**
		**Total sample**	**Only children with BMIz > -1.0**
SES indicators	Parent education level	-0.096**	-0.106**
	Parent employment status	0.014	0.028
General parenting	Nurturance	0.001	0.030
	Structure	-0.072^a^	-0.067
	Behavioral control	0.018	-0.044
	Overprotection	0.083*	0.091*
	Coercive control	-0.028	0.032

*Note:* Results of multivariate linear regression analyses; *p < 0.05, **p < 0.01; SES, Socio-Economic Status; Parent educational level, highest education attained, categorized into 1 = low level (10,8%; 8^th^ grade or less, attended some high school, technical school graduate), 2 = medium level (30,3%; high school graduate or general educational development, some college), and 3 = high level (58,9%; college graduate, post graduate study); Parent employment status, paid job, categorized into 1 = unemployed (12,4%), and 2 = employed (87,6%); ^a^This construct approached statistical significance (p = 0.051).

### SES indicator and child BMI differences in parenting dimensions

Furthermore, we assessed whether scores on the general parenting constructs differed depending on SES indicators and child BMI z-scores (results not reported in table). When performing an analysis of variance (ANOVA), parents in the highest category of education level scored significantly lower on overprotection (p < 0.01) compared to parents who scored in the middle or lowest category of education level (mean (SD) = 2.47 (0.52) vs. 2.61 (0.56) and 2.76 (0.63)) in the middle and lowest, respectively), and parents in the middle category of education level scored significantly lower on overprotection compared to parents in the lowest category of education level (p < 0.05). For coercive control, parents in the highest category of education level scored significantly lower on this construct compared to parents in the middle and lowest category of education level (p < 0.05; mean (SD) = 2.02 (0.49) vs. 2.10 (0.50) and 2.16 (0.57)). BMI z-scores significantly differed among children with parents in the highest category of education versus parents in the middle category of education (mean (SD) total sample including underweight children = -0.26 (1.32) vs. 0.07 (1.44), mean (SD) sample excluding underweight children = 0.34 (0.10) vs. 0.51 (0.78)).

Parents who were employed scored lower on the general parenting constructs of nurturance (independent samples *t*-test; p < 0.05; mean (SD) = 4.45 (0.40) vs. 4.53 (0.36)), behavioral control (p < 0.01; mean (SD) = 3.99 (0.49) vs. 4.10 (0.48)), and overprotection (p < 0.05; mean (SD) = 2.54 (0.55) vs. 2.62 (0.56)) compared to unemployed parents. BMI z-scores did not differ significantly among children with employed or unemployed parents.

### Questionnaire refinements based on quantitative and qualitative analyses

We started the development of the CGPQ with a 145-item instrument based on our parenting model, populated with existing items from previously developed questionnaires and refinement through author review meetings. Prior to data analysis, 30 items were dropped because of redundancy of item content or ambiguity. Based on the CFA and IRM analyses, 53 additional items were dropped. The resulting questionnaire (62 items) was reviewed again, with subsequent rewording of some items to improve clarity or simplify the language, and 23 additional items were added for better coverage of the sub-factors (excessive) monitoring and involvement; Hardy, Power and Jaedicke’s [77] modification of the Hetherington and Clingempeel’s [78] “Parent Assessment of Child Monitoring scale” and the “Protectiveness scale” developed by Hardy et al. [77] were used for this purpose. As a result of the author review, we elected to incorporate an additional sub-construct in the construct of behavioral control, i.e., “considering child input” (not being too strict to give a child space for personal development). This process resulted in an 85-item questionnaire representing the five parenting constructs and their corresponding sub-constructs each covered by five items.

To ensure that parents could comprehend the wording of the parenting items, the answer options and the instructions, five cognitive interviews were conducted in the Netherlands and the U.S., respectively. For the U.S. cognitive interviews, caregivers were recruited through the CNRC participant database. Families with eligible 5- to 13-year-old children, who previously indicated an interest in being contacted for studies, were identified and contacted. Baylor College of Medicine’s Institutional Review Board approved the study; all caregivers completed informed consent prior to data collection. A fifteen dollar gift card was provided to the caregiver for participation. For the Dutch cognitive interviews, participants also represented a convenience sample, recruited using personal network of the interviewer. The participants received a ten euro gift card for participation. For both countries, only minor changes were made in wording of items. Questionnaire completion time was about 15 minutes. Caregivers reported the instruction, items and answer options of the questionnaire were easy to understand and parents agreed that all aspects of parenting were covered. The current version of the questionnaire that resulted from the mixed-method approach as described above is incorporated in the online Supplement (Additional file 1) to this manuscript.

## Discussion

### Validation of the CGPQ

A parenting model, consisting of five constructs of parenting (i.e., nurturance, structure, behavioral control, overprotection, and coercive control) was used as the basis for the development of the CGPQ. CFA supported our five-factor model (moderately fitting) and together with IRM analyses helped us to reduce redundant items. The low reliability (a sample-dependent measure) of the “overprotection” parenting construct could be due to fewer number of items assessing this construct and possible heterogeneity of this construct in this sample.

Different approaches have been developed to conceptualize patterns of parenting, besides the typological approach to parenting. Whereas Maccoby and Martin [42] described authoritative parents high on two dimensions (responsiveness and demandingness), Steinberg [43] typified it by high levels on the dimensions of warmth and acceptance, psychological autonomy or democracy, and behavioral control. Grolnick and Pomerantz [79] tried to adapt the multiple-forms approach to defining parental control, by proposing that “only parenting characterized by pressure, intrusion and domination should be considered control, whereas parenting frequently labeled control but characterized mainly by guidance should be considered structure” (abstract, p. 165, see also [80]). However, this approach does not take into account the possibility of having different combinations of parenting and its multidimensionality [81], and all identified facets of the control construct [82]. Skinner et al. [24] identified three core dimensions in the assessment of parenting, each consisting of two opposing constructs: “warmth and rejection”, “structure and chaos”, and “autonomy support and coercion”, and supported the multidimensionality of these constructs. We suggest using latent class analyses or mixture modeling [83] for future studies using the CGPQ in order to assess the contribution and interaction of all five parenting constructs, which we propose will allow for better differentiation among parenting styles. As such, different combinations of the five parenting constructs may be used to characterize different clusters of parenting. This approach is supported in work of Grusec and Davidov [84], who imply that processes within each parenting domain are interacting with those in other domains.

### Parenting and personality

Confirming the findings of the meta-analytic review by Prinzie et al. [51], but also the recently conducted study of De Haan, Deković and Prinzie [85], this study showed that parent’s personality, in terms of the “Big Five”, was related to general parenting. Parents scoring high on the traits of extraversion, agreeableness, conscientiousness, and openness of experience also scored higher on positive aspects of parenting (i.e., nurturance, structure, behavioral control), as expected. Such parents generally provide supportive, structured and consistent home climates in which their parenting behaviors are expressed. These personality characteristics were generally inversely related to coercive control. Relationships of personality with overprotection were less pronounced. A reason for this might be that this construct was not covered by a wide range of items and reliability was low. Neuroticism (characterized by proneness to frustration, anger and distress) was indeed associated with low levels of nurturance and coercive forms of control, but also with chaotic home environments and overprotection.

### Parenting and child BMI

In our study, relationships between general parenting and child BMI z-scores were weak. A statistically significant effect for overprotection was found, indicating a potentially detrimental impact of overprotection on weight development. The pattern of associations between parenting constructs and child BMI z-scores confirmed theoretical assumptions (negative associations of child BMI with parental nurturance, structure and behavioral control and positive associations of child BMI with parental coercive control and overprotection), especially in the subsample excluding underweight children. Our study confirms the findings of previous studies in which also weak and potentially indirect effects of general parenting on weight status were found. To specify, Cislak, Safron, Pratt, Gaspar, and Luszczynska [52] conducted a systematic umbrella review and found that more general variables including general parenting constructs were found to have indirect and weaker effects on weight-related behaviors than more behavior-specific variables. Thus, general parenting is considered to be a more distal factor of actual child behavior than more proximal behavior-specific parenting practices [52]. The contextual influence of general parenting is likely to be more profound than its direct relationship with weight status or related behavior (dietary intake, physical activity, sedentary behavior) [5]. A major challenge for future empirical studies regarding child weight development will be to document under what conditions higher-order moderation is most or least likely to occur. More than a weak or absent effect of general parenting on child BMI, a contextual higher-order moderation approach is advocated to have surplus value in understanding the complex process of parent – child interactions in the area of childhood overweight.

### The contribution of SES indicators to parenting and child BMI

Parents with a high level of education were less likely to use overly forms of controlling parenting (i.e., coercive control and overprotection) and more likely to have children with lower BMI z-scores. In contrast, employed parents were less nurturing, structured and overprotective compared to parents who were unemployed. This could be because employed parents may have less time to spend with their children. Besides parent education level, child BMI is explained by overprotection and structure, although the latter was only marginally related to child BMI z-score. In future studies it is important to take into account the influence of socio-economic indicators on the relationship between parenting and child weight-related outcomes.

### Study limitations and strengths

Some limitations of the present study should be mentioned. First, it is likely that a bias occurred due to potential social desirability in reporting parenting behaviors, in particular as regards coercive forms of parenting. Additionally, self-reported BMI data may pose parents to underestimate their child’s weight and overestimate their child’s height. When performing secondary analyses on our data, we did find differences in mean scores on the parenting constructs of nurturance and structure between parents reporting on their child’s height and weight (*n* = 1015) and parents not reporting on these outcomes (*n* = 482). Parents with missing data or unrealistic values for height and/or weight for their child’s BMI scored significantly lower on both nurturance and structure compared to parents with complete and valid data for their child’s BMI (mean scores for nurturance: 4.42 (*SD* = 0.42) and 4.49 (*SD* = 0.39), respectively; mean scores for structure 3.78 (*SD* = 0.44) and 3.87 (*SD* = 0.45), respectively). It is likely that the present study yielded underestimates of associations between the scale scores of the CGPQ and child BMI z-scores, because of the parental reported nature of this study. Second, correlations with parent personality were examined using the reduced 62-item questionnaire and not the full 85-item questionnaire as this examination was part of the iterative development and validation process. Additionally, other indicators of parenting could have been included to assess construct validity, such as associations with similar parenting dimensions as measured by existing questionnaires using different items; observations of parenting; or reports from other family members. Demonstration of validity would be enhanced with performing a cross-validation, however, our sample was not sufficiently large to allow this. And lastly, caution is needed when generalizing these results as the samples might deviate from the general populations. A strength of our study is that we used a systematic mixed methods approach. We thoroughly searched the literature to develop our comprehensive general parenting model and identified questionnaires measuring each of our five parenting constructs. Based on advanced statistical analyses we assessed fit of our parenting model with a large sample of parents across three different countries and reduced questionnaire length.

### Future directions

Future work on the precursors and outcomes of parenting can benefit from measures that include all domains of parenting and make use of cluster-analytic approaches. Our questionnaire attempts to give such a comprehensive overview of parenting. Next steps include validation of the psychometric properties of the revised 85-item CGPQ and to assess its applicability to other target groups (adolescent self-reported parenting, and parent-reported parenting of infants and toddlers). Future directions should include studies that use the CGPQ across other cultural groups (e.g., Eastern cultures) without excluding important parenting constructs, to test for differential item functioning, factorial invariance and identify underlying universal characteristics of parenting that cut across cultures - characteristics that may differ in the way they are expressed in different cultures. Additionally, the contextual influence of parenting moderating the association between more specific parenting practices and children’s health outcomes [1] could be investigated more thoroughly. Also other variables including child temperament, child age, socio-economic status and culture are assumed to interact with parenting style, and should be taken into account in future research efforts.

## Abbreviations

BMI: Body mass index; CFA: Confirmatory Factor Analyses; CFI: Comparative Fit Index; CGPQ: Comprehensive General Parenting Questionnaire; CNRC: Children’s Nutrition Research Center; EAP/PV: Item separation reliability; IRM: Item-response modeling; NNFI: Non-normed fit index; RMSEA: Root Mean Square Error of Approximation; SES: Socio-economic status.

## Competing interests

The authors declare that they have no competing interests.

## Authors’ contributions

All authors contributed to the design of the study and were involved in the development of the questionnaire. ES conceptualized the study, performed recruitment of study participants and data collection, conducted the statistical analyses, and drafted the manuscript. KW provided statistical advice, and TP provided advice on the parenting literature and questionnaire. All authors participated in the interpretation of the results, reviewed draft versions of the manuscript and provided critical feedback. All authors have made a significant contribution to this manuscript, and all authors read and approved the final manuscript.

## Authors’ information

Kathleen B Watson is adjunct faculty of the Department of Pediatrics, Baylor College of Medicine/USDA Children's Nutrition Research Center. 

## Supplementary Material

Additional file 1 85-item Comprehensive General Parenting Questionnaire (caregivers of 5- to 13-year-olds). Click here for file
