# Supplementary material for: Development of the Comprehensive General Parenting Questionnaire for caregivers of 5-13 year olds
Source: Int J Behav Nutr Phys Act. 2014 Feb 10;11:15. doi: 10.1186/1479-5868-11-15 (PMC3926334; doi:10.1186/1479-5868-11-15)
Supplement: Additional file 1 — 85-item Comprehensive General Parenting Questionnaire (caregivers of 5- to 13-year-olds). [file 1479-5868-11-15-S1.doc]

**ONLINE SUPPLEMENT**

**85-item Comprehensive General Parenting Questionnaire (caregivers of 5- to 13-year-**olds)

| On the following pages you will see statements about parenting. We are interested in your opinion about these statements.  Please read all statements carefully. Sometimes there may be questions you think are not applicable to your family or child. Please try to answer these questions to the best of your ability. At times, there may be questions you might think: “I would like to act this way, but in reality I am not doing this”. Please answer these questions by indicating ***what you are actually doing***.  How much do you agree or disagree with the following statements?  (strongly disagree, somewhat disagree, neutral, somewhat agree, strongly agree) | | |
| --- | --- | --- |
|  | | **Measure** |
|  | **Nurturance – Autonomy support** |  |
| 1 | I encourage my child to be curious, to explore, and to question things | CRPR/PDI |
| 2 | I trust my child | PSCQ |
| 3 | I respect my child’s opinion and encourage him/her to express it | CRPR/PDI |
| 4 | I encourage my child to be true to himself/herself | PSCQ |
| 5 | I encourage my child to express his/her opinions even when I do not agree with him/her | PSCQ |
|  | **Nurturance – Social rewarding** |  |
| 6 | I praise my child when he/she does something good | DPQ a |
| 7 | I say something nice to my child as a reward for good behavior | CPBS a |
| 8 | When my child does his/her best, I praise him/her | GBPS a |
| 9 | I tell my child how much I appreciate it when he/she spontaneously helps me | CPBS a |
| 10 | I praise my child when he/she deserves it | DPQ a |
|  | **Nurturance –Responsiveness** |  |
| 11 | I know exactly when things are not going very well for my child | DPQ a |
| 12 | When my child is sad, I know what is going on with him/her | DPQ |
| 13 | I feel good about the relationship I have with my child | PSCQ |
| 14 | My child and I have warm affectionate moments together | CRPR a |
| 15 | I know exactly when my child has difficulty with something | DPQ |
|  |  | **Measure** |
|  | **Nurturance – Involvement** |  |
| 16 | I find time to talk with my child | POPS |
| 17 | I spend a lot of time with my child | POPS |
| 18 | I easily find a way to make time for my child | PASQ a |
| 19 | I attend as many of my child’s events and activities as possible | New item |
| 20 | I find it interesting and educational to be with my child for long periods | CRPR |
|  | **Structure – Inconsistent Discipline** |  |
| 21 | I have a hard time consistently enforcing rules with my child | New item |
| 22 | I do not always follow through when I threaten to discipline my child | New item |
| 23 | I threaten discipline more often than I actually give it | CRPR a CPBS a |
| 24 | When I discipline my child, I sometimes end the punishment early | CPBS a |
| 25 | There are times I just do not have energy to make my child behave as he/she should | PDI |
|  | **Structure – Consistency** |  |
| 26 | When I tell my child I will do something, I do it | PSCQ |
| 27 | I use clear and consistent messages when I tell my child to do something | New item |
| 28 | I try not to change the rules at home very often | PSCQ a |
| 29 | I try not to forget the promises I make to my child | CRPR a |
| 30 | I explain the reasons behind our family rules | New item |
|  | **Structure – Organization** |  |
| 31 | I make sure my child has enough time to get ready for school | New item |
| 32 | I help my child schedule time for household chores | New item |
| 33 | I help my child plan his/her activities for the day/week | New item |
| 34 | I teach my child to keep his/her bedroom clean and orderly | New item |
| 35 | I make sure my child is at school on time | New item |
|  |  |  |
|  |  |  |
|  |  |  |
|  |  |  |
|  |  | **Measure** |
|  | **Structure - Scaffolding** |  |
| 36 | When my child faces a difficult problem, I help him/her break it down into smaller steps | New item |
| 37 | When I talk with my child about his/her problems, I really try to help him/her | DPQ |
| 38 | I put time and energy into helping my child, when he/she asks for it | PPOS a |
| 39 | When my child has difficulties, I help him/her | DPQ |
| 40 | When my child has a problem, I help him/her figure out what to do about it | PSCQ |
|  | **Behavioral Control – Monitoring** |  |
| 41 | I keep track of my child’s activities with friends | PACMS a |
| 42 | I pay attention to where my child is | GPBS a |
| 43 | I watch my child to make sure he/she behaves appropriately | PRS (M) |
| 44 | I am aware of what my child is doing when he/she is at home | PACMS |
| 45 | I am aware of my child’s choice of friends, who they are, what they are like | PACMS |
|  | **Behavioral Control – Maturity demands** |  |
| 46 | I expect my child to follow our family rules | PSCQ |
| 47 | I have clear expectations for how my child should behave | PRS (E) a |
| 48 | I require my child to behave in certain ways | PRS (E) |
| 49 | I make sure that my child understands what I expect of him/her | PSCQ a |
| 50 | I teach my child to follow rules | PRS (E) a |
|  | **Behavioral Control – Non-intrusive discipline** |  |
| 51 | When I correct my child’s behavior, I explain why | PSCQ |
| 52 | When my child goes against a rule I take away a privilege | New item |
| 53 | I correct my child when he/she breaks the rules | New item |
| 54 | I correct my child’s minor misbehaviors with explanations | New item |
| 55 | I would ground my child if he/she committed a serious offense | New item |
|  |  |  |
|  |  |  |
|  |  |  |
|  |  |  |
|  |  | **Measure** |
|  | **Behavioral Control – Considering child input** |  |
| 56 | I want my child to always obey me *(reversed coding)* | PDI a |
| 57 | I place a lot of emphasis on obedience in my child *(reversed coding)* | PDI a |
| 58 | If I give my child too many rules, he/she will grow up to be a unhappy adult | PDI a |
| 59 | I make sure I give my child lots of freedom to make mistakes and learn from them | PDI a |
| 60 | I give my child a lot of freedom to make up his/her own mind | PDI a |
|  | **Coercive control – Psychological control** |  |
| 61 | When my child does something that is not allowed, I do not talk to him/her until he/she says he/she is sorry | GPBS |
| 62 | I am less friendly with my child if he/she does not see things my way | PCS |
| 63 | I make sure my child is aware of how much I sacrifice for him/her | CRPR a |
| 64 | I make my child feel guilty when he/she does not meet my expectations | Olsen (2002)  p. 246 |
| 65 | When my child hurts my feelings, I stop talking to him/her until he/she pleases me again | PCS |
|  | **Coercive control – Physical punishment** |  |
| 66 | I spank my child when he/she does not obey rules | GPBS |
| 67 | I spank my child when he/she does something wrong | GPBS a |
| 68 | I spank my child when he/she is disobedient | GPBS a |
| 69 | I use physical punishment to discipline my child | CRPR a |
| 70 | I spank my child when he/she is behaving inappropriately | New item |
|  | **Coercive control – Authoritarian control** |  |
| 71 | I teach my child to stay in control of his/her feelings at all times | CRPR a |
| 72 | I do not allow my child to question my decisions | CRPR/PAQ |
| 73 | When I ask my child to do something, I expect him/her to do it immediately without any questions | PAQ |
| 74 | I let my child know that I am the boss in our house | PAQ a |
| 75 | I do not allow my child to get angry with me | CRPR |
|  |  |  |
|  |  | **Measure** |
|  | **Overprotection – Excessive monitoring** |  |
| 76 | I am always aware of what my child is doing | New item |
| 77 | I let my child play a lot by himself/herself without my supervision *(reversed coding)* | CRPR a |
| 78 | When my child has a friend over, I frequently check to see what they are doing | New item |
| 79 | I make sure I know exactly where my child is at all times | CRPR a |
| 80 | I prefer my child play at our house with his/her friends rather than playing at a friend’s house | PQ |
|  | **Overprotection – Excessive involvement** |  |
| 81 | Every free minute I have I spend with my child | DPQ |
| 82 | I always help my child with everything he/she does | DPQ a |
| 83 | When my child has lost something, I stop what I am doing to find it before he/she gets too upset | PQ a |
| 84 | I do not let my child get involved in activities or tasks where he/she may potentially fail | PQ a CRPR a |
| 85 | I carefully plan my child’s day so that he/she has enough activities to keep him/her busy | PQ a |

*Note*. Copyright Sleddens, O’Connor, Watson, Hughes, Power, Thijs, De Vries, Kremers. This questionnaire is also available in the Dutch and Spanish language. Please contact Ester Sleddens at Ester.Sleddens@maastrichtuniversity.nl if you are interested in using or adapting the questionnaire for your research. Please note that the sub-construct of ‘Considering Child Input’ (Behavioral Control) is not previously tested before using CFA and IRM.

Abbreviations:

CRPR: Child Rearing Practices Report

DPQ: Dutch Parenting Questionnaire (‘Nijmeegse Opvoedingsvragenlijst’)

GPBS: Ghent Parental Behavior Scale

PAQ: Parental Authority Questionnaire

PASMS: Parental Assessment of Child Monitoring Scale

PCS: Psychological Control Scale

PDI: Parenting Dimensions Inventory

PPOS: Perceptions of Parents Scales

PRS (EB): Parental Regulation Scale, parental expectations for behavior scale

PRS (M): Parental Regulation Scale, parental monitoring of behavior scale

PSCQ: Parents as Social Context Questionnaire

PQ: Protectiveness Questionnaire
